# Global health initiative investments and health systems strengthening: a content analysis of global fund investments

**DOI:** 10.1186/1744-8603-9-30

**Published:** 2013-07-26

**Authors:** Ashley E Warren, Kaspar Wyss, George Shakarishvili, Rifat Atun, Don de Savigny

**Affiliations:** 1 Department of Epidemiology and Public Health, Swiss Tropical and Public Health Institute, Socinstrasse 57, 4002, Basel, Switzerland; 2 University of Basel, Petersplatz 1, 4003, Basel, Switzerland; 3 Swiss Centre for International Health, Swiss Tropical and Public Health Institute, Socinstrasse 57, 4002, Basel, Switzerland; 4 The Global Fund to Fight AIDS, Tuberculosis and Malaria, Geneva, Switzerland; 5 Imperial College London, South Kensington Campus, London SW7 2AZ, UK; 6 Department of Global Health and Population, Harvard School of Public Health, Harvard University, Cambridge, USA

## Abstract

**Background:**

Millions of dollars are invested annually under the umbrella of national health systems strengthening. Global health initiatives provide funding for low- and middle-income countries through disease-oriented programmes while maintaining that the interventions simultaneously strengthen systems. However, it is as yet unclear which, and to what extent, system-level interventions are being funded by these initiatives, nor is it clear how much funding they allocate to disease-specific activities – through conventional ‘vertical-programming’ approach. Such funding can be channelled to one or more of the health system building blocks while targeting disease(s) or explicitly to system-wide activities.

**Methods:**

We operationalized the World Health Organization health system framework of the six building blocks to conduct a detailed assessment of Global Fund health system investments. Our application of this framework framework provides a comprehensive quantification of system-level interventions. We applied this systematically to a random subset of 52 of the 139 grants funded in Round 8 of the Global Fund to Fight AIDS, Tuberculosis and Malaria (totalling approximately US$1 billion).

**Results:**

According to the analysis, 37% (US$ 362 million) of the Global Fund Round 8 funding was allocated to health systems strengthening. Of that, 38% (US$ 139 million) was for generic system-level interventions, rather than disease-specific system support. Around 82% of health systems strengthening funding (US$ 296 million) was allocated to service delivery, human resources, and medicines & technology, and within each of these to two to three interventions. Governance, financing, and information building blocks received relatively low funding.

**Conclusions:**

This study shows that a substantial portion of Global Fund’s Round 8 funds was devoted to health systems strengthening. Dramatic skewing among the health system building blocks suggests opportunities for more balanced investments with regard to governance, financing, and information system related interventions. There is also a need for agreement, by researchers, recipients, and donors, on keystone interventions that have the greatest system-level impacts for the cost-effective use of funds. Effective health system strengthening depends on inter-agency collaboration and country commitment along with concerted partnership among all the stakeholders working in the health system.

## Key messages

• 37% (US$ 362 million) of funding in Round 8 of the Global Fund to Fight AIDS, Tuberculosis and Malaria was for health system strengthening.

• Of the HSS funding, 38% (US$ 139 million) was for system-level interventions while 62% (US$ 223 million) was dedicated to disease-specific system strengthening activities.

• Around 82% (US$ 296 million) of health systems strengthening funding in Round 8 was dedicated to service delivery, human resources, and medicines.

## Background

In less than 20 years approximately 100 global health initiatives (GHIs) have been created to meet the Millennium Development Goals (MDGs). These GHIs, often established as public-private partnerships, have leveraged and mobilised unprecedented levels of funding channelled through governments and civil society organizations for specific diseases and targeted interventions [1,2]. At country level this has led to a fragmentation of service delivery with unpredictable effects on health systems [3-7]. Investment in health system strengthening (HSS) was proposed to mitigate adverse effects of the increasingly complex funding flows [3,8-13], address health system bottlenecks, and accelerate progress towards the MDGs [8,10,14-17].

The Global Fund to Fight AIDS, Tuberculosis and Malaria (Global Fund), the Global Alliance for Vaccines and Immunisation (GAVI), and the President’s Emergency Plan for AIDS Relief (PEPFAR), for example, channel funds for HSS based on the World Health Organization (WHO) health systems framework that identifies ‘six building blocks’ [7,18-21]: governance, financing, information, human resources, medicines and technology, and service delivery [21,22]. This framework has been discussed extensively and many derivatives have been developed for describing and studying health systems [2,7,8,18,23-30]. Because GHIs that support strengthening of recipient systems often use the WHO framework as the foundation of their HSS programs, the building blocks can be useful for cross-analysis of donor funding.

Our study, which aimed to quantify and categorize the Global Fund’s HSS funding, had two objectives: 1) to propose an adapted, operational framework with which to classify GHIs’ financial investments in HSS, and 2) to apply it to the Global Fund.

Although there is a body of literature on disease-specific investments by GHIs and their impact [8,31-35], there are relatively few publications on HSS-specific funding. Meanwhile, there has been substantial criticism that the Global Fund and other GHIs allocate insufficient resources to HSS [26,36-39]. This is likely a reflection of the debate *within* these global initiatives on the level of funding that should be allocated to HSS and whether the funding should be through disease-specific interventions or through more generic investments in health systems to benefit target diseases and beyond [18,40]. The Global Fund allows investment in health systems both through disease-specific funding and through general health system strengthening activities [7,41]. Over the years the Global Fund has used a range of approaches to fund HSS activities. For example, in 2006 there was a specific call for HSS grants in Round 5 [42], and ‘cross-cutting’ health system interventions could be funded in subsequent Rounds [42,43]. Though the Global Fund provides an overview of their HSS financing [44], few studies have attempted to estimate the proportion of funding specifically allocated to health systems strengthening through disease-specific and/or to more generic (cross-cutting) interventions [17].

In 2010, the Global Fund provided approximately two thirds of international funding for fighting malaria and tuberculosis (TB) and nearly a quarter of funding for AIDS; claiming also to be the “largest multi-lateral channel for efforts to strengthen health systems” [45]. In 2011 the Global Fund provided 10% of total development assistance for health [13]. On 1 March 2008, the Global Fund launched Round 8. By 8 November 2008, 140 grants were approved for 65 low- and middle-income countries with a total value of approximately $2.5 billion for *Phase I* (almost twice as large as earlier Rounds). The largest single grant in Round 8 was awarded to Ethiopia for malaria with a value of approximately $133 million (5.4% of overall), and the smallest grant, worth approximately $532 000, was awarded to Tunisia for TB. Overall, Nigeria received the largest total amount of $340 million (~14% of the total for Round 8) through four grants – three dedicated to malaria and one dedicated to HSS.

When adapting the existing WHO health system framework [21], we considered other examples developed by Biesma et al. [8], Samb et al. [2], and Shakarishvili et al. [17]. The framework by Biesma et al. includes assessment of the Governance, Human Resources, and Financing building blocks, but not for activities in the Medicines and Technologies, the Service Delivery, or the Information subsystems - though monitoring and evaluation (M&E) is included [8]. The framework by Samb et al. is more comprehensive and is based on the WHO building blocks, but it was developed for assessing outcomes/effects rather than investment amounts [2]. The framework developed by Shakarishvili et al. was developed at the Global Fund specifically for tracking HSS investments using four domains of Stewardship and Governance, Health Services, Financing, and Monitoring and Evaluation (including health information systems) [17]. We built upon these frameworks to elaborate a classification mirroring the WHO six building blocks as many countries used the six building blocks framework when requesting funds from the Global Fund.

## Methods

We systematically reviewed the grants funded by the Global Fund in Round 8 – the largest Round totalling approximately $2.5 billion for *Phase I*. We used a framework (See Table 1 for abridged version; Additional file 1: Table S1 for full version) that draws on the WHO six building blocks [21], frameworks developed by Biesma et al., Samb et al., and Shakarishvili et al., the WHO’s Framework for Action, *Fixing Health Systems*, and *Systems Thinking* by de Savigny and Adam, and discussions with experts in the respective fields [2,8-10,16,17,21]. The adapted framework we propose provides a comprehensive (but not exhaustive) framing of system-level interventions requested by countries and funded by the Global Fund. Such a framework is meant to serve as a tool for cataloguing and mapping the HSS content and funding of any Global Fund proposal. Besides determining the monetary amount invested in each building block, analysis with this framework serves to determine whether or not health system strengthening is restricted to disease programmes or is more generic.

**Table 1:** **HSS funding assessment framework** (**abridged**)

***Building Block***	***Function***	***Intervention***
***Governance***	Capacity building	See Additional file 1: Table S1
	Harmonisation	
	Sector integration	
	Decentralisation	
	National health strategy development	
	Coordination	
***Financing***	Maximise social protection	
	Improve resource effectiveness	
	Patient and/or provider incentives	
	Financial management transparency	
***Information***	Health information systems strengthening	
	Strategies to increase evidence-based planning	
	Increase accessibility of information	
***Human resources***	Support for pre-service training	
	Support for in-service health workforce	
***Medicines and technology***	Support for rational use of essential medicines	
	Improve management of essential medicines	
	Affordable, quality essential drugs programme	
	Health service supplies (non-consumables)	
***Service delivery***	Infrastructure	
	Measures to increase coverage - supply	
	Measures to increase coverage - demand	

The first tier in the HSS Funding Assessment Framework is comprised of the WHO-defined health system *building blocks*. The second tier contains relevant *functions* of each building block, and at the third tier – the deepest level of resolution – describes system-level *interventions* (only the first two tiers are shown in Table 1). In the rare occasion that a budgeted activity (i.e. action having its own budget line in the detailed budgets provided to the Global Fund by the Principal Recipient) could not be classified at the *intervention* level, it was classified at the next highest level of resolution, either the second or first tier.

All grant materials were collated from the Global Fund internal database, with the consent of the Strategy, Performance, and Evaluation Cluster at the Global Fund. Overall 139 grants were funded in Round 8 including 52 grants dedicated to HIV/AIDS, 34 to tuberculosis, 46 to malaria, and 7 to HSS.

Of the 139 grants funded in Round 8, 27 were excluded from classification due to language (submitted in either French or Spanish) and budgets from 41 grants were unavailable at the time of analysis. We selected 52 grants from the remaining 71 grants; this sub-set is representative of the Round 8 grant portfolio in terms of region and disease-component (using chi-squared analysis; see Additional file 1: Tables S2, S3, and S4). The sub-set of grants is also representative of the Round 8 portfolio in terms of dollar-value. The median dollar (US$) value of grant agreements in Round 8 is US$8.5 million with an Interquartile Range (IQR) of US$22.9 - 4.4 million. The median dollar value of the study subset is US$9.8 million with an IQR of US$23.3 - 4.9 million.

Additional file 1: Table S1 of the Signed Grant Agreement, which outlines key activities, was analysed to establish the aims of each grant; the SGA’s main body of text was consulted for clarification when necessary. The Final Detailed Budget for *Phase I*, included in the grant agreement, provided to the Global Fund by the Principal Recipient, was reorganized to fit a standardised template in Microsoft Excel to remove any inconsistencies in formatting and, therefore, facilitate comparison. For every grant the grant number, region, agreed amount, and disease component, as well as the detailed description of each activity, Cost Category, and the Service Delivery Area (as assigned by the Principal Recipient) were included for accurate classification. Budget lines dedicated to activities for *Phase II* were excluded. Our calculations are based on the Sum Total found in the grant budget.

Upon standardization of the Final Detailed Budget in Microsoft Excel, three analysis-specific columns were added for classification using the HSS Funding Assessment Framework, tagging the activity as disease-specific or system-wide, and categorizing non-HSS activities. Each cell in the ‘HSS Classification’ column contained a drop-down menu with the HSS Funding Assessment Framework, and each cell in the ‘Disease-Specificity’ column contained a drop-down menu for differentiating between disease-specific and system-level classification. Each budget was then classified line-by-line with these two categories. If a budget line was deemed “Non-HSS”, it was tagged and classified using the drop-down menu containing “Grant Management”, “Salary / Per Diem”, “Commodities, Diagnostics, and Drugs”, or “Other”. A ‘Comments’ column was used for acronym definitions and notes or rationale about classification. For example, the activities supporting the development of a Malaria Surveillance Database in Swaziland were categorized as *burden of disease data collection* rather than *design and development of HMIS*. The corresponding ‘Comments’ column contained a note explaining why it was not classified as HMIS strengthening: “data is not routine nor is it gathered at the health facility level”.

**Figure 1:**
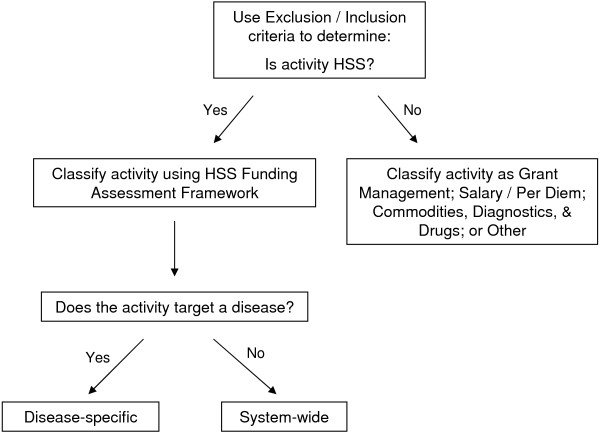
Decision tree used in classification of each budgeted activity.

In summary, each activity was examined using the process outlined in Figure 1.

An activity was considered “disease-specific” if it followed a conventional ‘vertical-programming’ approach - targeting system support within one or more building blocks specifically for AIDS, tuberculosis, and/or malaria. An activity was deemed “system-wide” if it explicitly targeted any building block, either individually or collectively, *without* explicitly targeting one of the three diseases [10].

Exclusion/Inclusion criteria were developed to distinguish between HSS and Non-HSS budgeted activities (see Additional file 1: Table S5). Stand-alone routine activities, such as salaries, meetings, rent, and ongoing operating costs, used to *maintain* rather than *strengthen* the health system were excluded. Although these are important activities fundamental to any health system, they do not improve or reinforce *how* the system functions nor do they alter the effectiveness or efficiency of the health system. If, however, they were nested within an HSS activity, then they were classified accordingly. For example, Swaziland’s budget-line “Technical assistance – Malaria Surveillance Database upgrade” that was nested within the activity “Development of Malaria Surveillance Database” was labelled as disease-specific HSS. Funding for training or support for persons working outside of the health system, i.e. anyone whose primary intent is anything other than improving or maintaining health (ex. social workers, police officers, etc.) was also excluded. The support of international technical assistance was also excluded because funding for temporary international consultants accrues not to local staff or the health system budget. Incentives for patients framed as patient support were excluded because the target is not the system but rather the patient.

Upon completing the classification of the 7,261 budgeted activities, the data were filtered and tabulated by region, *building block*, *function*, *intervention*, disease-specificity, and each aforementioned Non-HSS tag. The currencies were standardized to USD. Original grant agreements signed using the Euro (€) were converted to USD based on the start date of the grant agreement [46]. Start dates ranged from 1 July 2009 to 1 October 2010 (Additional file 1: Table S6 for dates and corresponding exchange rates).

## Results

**Figure 2:**
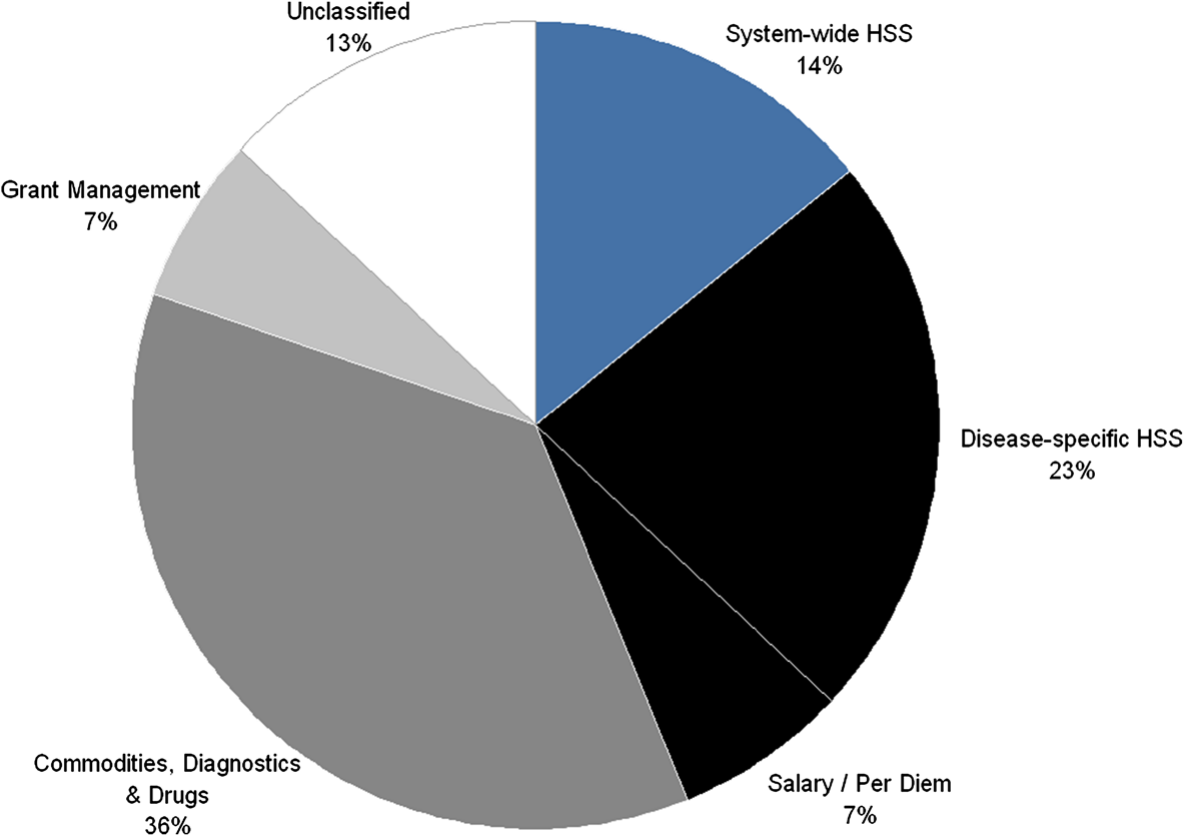
**Resource allocation profile of HSS and Non-HSS activities.** Sub-set absolute values- Overall $1.1 billion; Disease-specific HSS $223 million; System-wide HSS $184 million; Grant Management $66 million; Salary / Per Diem $67 million; Commodities, Diagnostics, and Drugs $358 million; and Unclassified $129 million.

Around 37% of the activities in our sample (approximately US$362 million) qualified as health system strengthening interventions according to the HSS Funding Assessment Framework. Of these HSS activities 38% (~US$139 million) were categorized as system-wide strengthening whereas 62% (~US$223 million) were considered to be disease-specific HSS activities (with an *overall* proportion of 14% (US$ 139 million) and 23% (US$ 223 million); respectively (Figure 2). Of the Non-HSS activities Commodities, Diagnostics, and Drugs received the most funding at 36% (~US$358 million) of the total.

**Figure 3:**
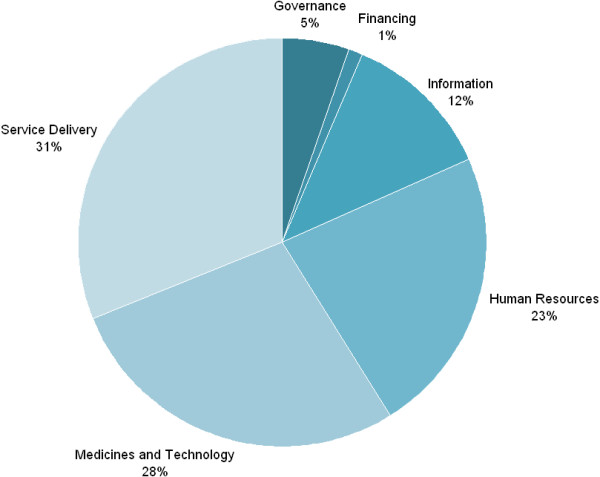
**Resource allocation profile of WHO building blocks.** Sub-set absolute values- Total HSS $463 million; Governance $19 million; Financing $4 million; Information $44 million; Human Resources $82 million; Medicines and Technology $101 million; and Service Delivery $112 million.

Nearly one-third of the funds budgeted for HSS were dedicated to Service Delivery, whereas the Governance, Financing, and Information *building blocks* comprise under one-quarter of the funds (Figure 3).

Table 2 provides higher resolution to the division of resource allocation within each *building block*. The Governance, Financing, and Human Resources *building blocks* are heavily skewed towards a primary function, but the Information, Service Delivery, and Medicines and Technology *building blocks* have benefited from more even investments across multiple *functions*.

**Table 2:** Proportion of funding per WHO building block

***Health system building block interventions***	***% of spending within building block***		
	**Disease-specific**	**System-wide**	**Total**
**Governance**			
Sector integration	67	40	**55**
Decentralisation	5	21	**12**
Capacity building	1	25	**11**
National health strategy development	14	5	**10**
Coordination	9	3	**6**
Harmonisation	5	3	**4**
**Financing**			
Patient and/or provider incentives	99	86	**93**
Improve resource effectiveness	0	13	**6**
Financial management transparency	1	0	**1**
Maximise social protection	0	1	**0**
**Information**			
Health information systems strengthening	39	74	**55**
Strategies to increase evidence-based planning	54	18	**37**
Increase accessibility of information	7	3	**5**
**Human Resources**			
Support for in-service health workforce	99	93	**97**
Support for pre-service health workforce	1	7	**3**
**Medicines and Technology**			
Health service supplies (non-consumables)	25	90	**54**
Improve mgmt of essential medicines	55	9	**34**
Support for rational use of essential medicines	19	1	**11**
Affordable, quality EDP	1	1	**1**
**Service Delivery**			
Infrastructure	38	74	**49**
Measures to increase coverage - demand	55	19	**44**
Measures to increase coverage - supply	7	6	**7**

EDP: Essential drugs programme.

### Governance

The Governance *building block* received 5% (~$19 million USD) of the HSS resources. Over half the funds dedicated to the Governance *building block* was for ‘sector integration’ , with a small amount (~1%, combined) for ‘coordination’ and ‘harmonization’ *functions*.

Activities devoted to collaboration with civil society account for approximately 87% (US$ 9 million) of the funding dedicated to ‘sector integration’ (and therefore almost 48% of the funds for Governance; just over US$9 million). Within the Governance *building block* all the funds dedicated to training on sector integration, decentralization of management/resource control, and measures to increase accountability/transparency were implemented through activities that were deemed system-wide. All activities for collaboration between Ministries and the decentralization of leadership/ownership were disease-specific. Examples include “[*Ministry of Health*], [*Ministry of Youth and Sports*], [*Ministry of Education*] *and NGO staff would do outreach to all vulnerable youths populations and provide them with HIV*/*AIDS preventive services*” in Mauritius and “*Leadership and coordination of county*-*level leading groups for TB control among the migrant population*” in China; respectively.

### Financing

Around one percent (~US$4 million) of Round 8 resources were allocated to the Financing *building block*, and of this more than 93% (~US$3.7 million) was dedicated to ‘patient and/or provider incentives.’ Support for ‘financial management transparency’ and ‘maximizing social protection’ received negligible funding, and *interventions* classified under the “improving resource effectiveness” *function* received approximately 6% (US$ 237 000). Of the six *building blocks* Financing had the greatest skewing for few *interventions*. There was greater heterogeneity in system-wide *interventions* than in disease-specific. All funds classified as disease-specific fell within ‘patient and/or provider incentives’ and ‘financial management transparency’ while all four *functions* were supported (albeit minimally) with system-wide *interventions*.

### Information

Global Fund Round 8 spending on the Information *building block* was around 12% (~US$44 million); of this, 55% (~US$24 million) was dedicated to the *function* ‘health information system strengthening’. All funds for introducing an electronic records system were through system-wide activities such as “*develop and implement electronic medical record systems in 3 central hospitals*” in Mozambique and Bulgaria’s “*online data base with the health facilities and professionals trained and involved in* [*Planning*, *Public Policy*, *and Management*]”. The dominant *interventions* were: training/compensation of staff (24%; US$ 10 million) and M&E strengthening (25%; US$ 9 million). Harmonization with the national health information system (HIS), design and development of health management information system, and standardization/rationalization of indicators – all important *interventions* – received in combination less than 6% (US$ 805 000) of the funds allocated to Information *building block*. The resources dedicated to design and development of HMIS and the standardization/rationalization of indicators all fell under system-wide spending. Examples include Nigeria’s “*strengthen routine data generation and flow from public*/*private facilities and community based health providers for the National Health Management Information System*” and a “*workshop to develop a comprehensive list of indicators for reporting on the health system*” in Sudan.

### Human resources

Approximately 23% (~US$82 million) of HSS spending was dedicated to Human Resources. Around 97% (~US$80 million) of Human Resources investments were dedicated to ‘support for in-service workforce’. The top three *interventions* in the Human Resources *building block* were continuing education/on-the-job training, leadership and management capacity building, and improvement of feedback and supervision (56% - US$ 46 million, 27% - US$ 23 million, and 10% - US$ 8 million; respectively). Task shifting *interventions* such as “*malaria control work load analysis*,” and support for referral system received fewer funds and were funded entirely for disease-specific purposes whereas Salary/Per Diems (Non-HSS) received 7% of support overall. All funds dedicated to supporting training institutions was for system-wide activity. Overall, 66% (~US$54 million) of support for the Human Resources *building block* was for disease-specific *interventions*.

### Medicines and technology

Approximately 28% (~US$101 million) of HSS spending was dedicated to Medicines and Technology. Just over 50% (US$ 54 million) of this was for the provision/maintenance of equipment and close to 26% was for equipping central and regional medicine depots (~US$54 million and ~ US$26 million; respectively). Both of these activities made it in the top ten of total expenditure overall. Overall, the provision/maintenance of equipment was the largest system-wide expenditure. Strengthening of supply chain management received 8% (US$ 8 million) of the support provided to Medicines and Technology, and of this 98% (US$ 7.9 million) was dedicated to disease-specific *interventions*, for example, to “*improve supply chain management for HIV test kits*, *and other medical supplies necessary to conduct HIV test and counselling at all health care facilities offering the services*” in Zimbabwe and “*logistic for storing and distribution* [*insecticide treated nets*] *to target municipalities*” in Brazil. Strengthening procurement systems was entirely through system-wide measures, though this *function* represents less than 1% (US$ 472 000) of the *building block*.

### Service delivery

Over 30% (~US$112 million) of Global Fund’s HSS resources were dedicated to Service Delivery. Within this, 49% (US$ 55 million) was dedicated to ‘infrastructure’ and 44% (US$ 49 million) to ‘measures to increase coverage’ from the demand side (~US$55 million and ~ $49 million). Of the ‘measures to increase coverage’ from the supply side, 25% (US$ 2 million) was dedicated to disease-specific standardization of care; “*establish systems for early infant diagnosis for all HIV exposed babies*” in Ghana, for example. The three most common *interventions* for ‘infrastructure’ were provision/maintenance of transportation, maintenance of institutions/dispensaries, and support for waste management systems. Very little funding was dedicated to capital construction or maintenance of storage facilities.

Most of the funding dedicated to ‘measures to increase coverage - demand” was channelled through activities for social marketing to increase awareness. A much smaller share was for alignment of services with cultural norms. Social marketing to increase awareness is in the top ten in terms of overall funding. Approximately 99% (US$ 31 million) of the financing for social marketing had disease-specific priorities. Social marketing to increase awareness about primary health care services or on general health was negligible.

## Discussion

The HSS Funding Assessment Framework used for this study was developed to track, compare, and understand financial *inputs* for health systems by global health initiatives and applied to the Global Fund Round 8 [16,38]. Activities were classified using signed *Phase I* budgets, not effective disbursements or expenditure. Most programs do not manage to absorb the entire budget, but budgets represent perceived priorities of the Principle Recipient. Each budgeted activity was classified along a building block rather than expected downstream *outcomes*; due to the non-linear interactions between building blocks one cannot attribute a given input to a given output. For example, Bulgaria’s “*Training Roma community workers in outreach work and needs assessment of clients*” was classified as ‘continuing education/on-the-job training’ and cannot directly credited to a given outcome such as access, equity, or quality.

Exclusion/Inclusion criteria were developed before the analysis during the reading of randomly selected Signed Grant Agreements and Original Proposals from Round 8 (see Additional file 1: Table S5). The inclusion criteria served more as a reminder for classifying a few frequently budgeted activities; the HSS Funding Assessment Framework (see Additional file 1: Table S1) is fairly comprehensive, and therefore obviated the need to establish extensive inclusion criteria.

One limitation to this first application study is the lack of a validity test for either the framework or the Exclusion/Inclusion criteria. Only one person reviewed the grants and classified activities. This retrospective analysis could be strengthened if there were two reviewers. The budgets are reviewed by the Global Fund before approval, so overall they are quite clear, but it could be useful if the reviewers were in contact with Principle Recipients to discuss and clarify specific budget lines if need be. Ideally, in order to avoid placing any further strain on Recipients and further fragmenting reporting systems such tracking/analyses could be incorporated into routine auditing. Guidelines could be developed for recipients and they could classify each activity themselves when designing the budget.

There are other inherent limitations to this study based on the Global Fund documents used. However our overall estimate of HSS support in Round 8 (37%) concurs with that of the GF’s own assessment (cited on website [44] on 24 January 2011 at the time of analysis).

The HSS Funding Assessment Framework could also potentially be applied to other GHIs supporting HSS activities, for example the GAVI Alliance and the PEPFAR, depending on the resolution of approved budgets. The GAVI Alliance uses HSS as a means to reach their immunization-specific objectives [1,47]. This framework could capture the landscape of their investments as they utilize the WHO definition for health system and its four key functions [48]. It uses the WHO six building blocks, however, they are rephrased to fit into immunization program functions [38].

PEPFAR, is primarily interested in HSS activities as a means for ensuring a sustainable response to the HIV/AIDS pandemic. Its primary HSS activities include task shifting; and training, retaining, and creating support systems for health workers. Unfortunately, PEPFAR engages in bilateral partnerships that often circumvent the recipient health sector’s existing structures [49]. It is unclear what explicit HSS approach or framework PEPFAR uses; they avoid much of the HSS rhetoric in the literature they produce [50]. Thus, the use of such a framework could be particularly useful in elucidating specific PEPFAR HSS investments.

### Governance

Arguably, governance is the cornerstone of a health system, as it includes the formal and informal players that define and enforce rules needed for the system to perform its key functions of promoting and protecting the health of its population [51,52]. Recently frameworks have been developed specifically to assess governance in health systems [53,54]. Though they include activities outside the scope of GHIs, these more exhaustive frameworks include many relevant principles: transparency, accountability, equity and inclusiveness, provision of information, standards, and regulations, as well as the importance of relevant stakeholder participation, including civil society. In general, there is a gap in the literature about specific governance interventions in the health system. Therefore there are no concrete guidelines for strengthening governance mechanisms.

Overall, collaboration with civil society is a distinguishing feature of GHIs, and the Global Fund in particular, so it is not surprising to find direct evidence of heavily weighted investment in civil society organizations. There was support for a wide range of activities to engage civil society. Lesotho prioritized sustained institutional capacity, sector-wide representation of civil society constituencies, participation in regional and continental civil society networks, and outlining laws and policies needed for civil society strengthening. Mauritius focused specifically on engaging prison staff for developing national protocols and guidelines for needle exchange and condom distribution. Tajikistan requested funds for community outreach strategies based on multi-stakeholder round table discussions.

Although there is evidence that the voice of the civil society component of the Country Coordinating Mechanism is minimized in the outline of program priorities [55], it appears that the Global Fund is dedicated to increasing outlets for civil society engagement. Researchers have found there to be both positive and negative effects of high levels of civil society engagement. Civil society has played an important role in expanding service delivery to include marginalized groups, monitoring good governance and increasing responsiveness to community health priorities, advocating for evidence-based health policy reforms, and providing guidance for patient follow-up and outreach services [56-62]. However, civil society organizations are sometimes considered to have inadequate levels of accountability, transparency, and legitimacy [63,64].

Given the allegations of corruption uncovered by the Global Fund’s Inspector General in some Principal Recipients and sub-recipients, we note a surprisingly low proportion of funds dedicated to measures aimed at increasing accountability/transparency (~1% -US$ 190 000 - of funds allocated to Governance). Only three grants contained such interventions. Liberia budgeted for monitoring and supervision support; Lesotho proposed to establish a Secretariat within an HIV/AIDS cross-sectoral program, and Serbia sought to develop guidelines for supervision within their HIV/AIDS program. It would be encouraging to see increased measures of accountability/transparency accompany activities that involve collaboration between the Principal Recipient and external forces such as civil society, various Ministries, and other sectors.

It is also notable that minimal funding is allocated to the ‘harmonization’ *function* (approximately 4% (US$ 773 000) considering the evidence of increased coordination and alignment between some GHIs and recipient governments / country-level sectors [35]. Harmonization between donors and governmental agendas is important for ensuring that recipient priorities are being addressed. It could be that over time the Global Fund aligns with national priorities through continued efforts to collaborate with civil society who in turn advocate at the national level.

One budgeted activity that could only be resolved at the second-tier of the HSS Funding Assessment Framework, but is a good example of a system-wide activity in the Governance *building block*, was found in a grant from Tajikistan. The activity was described as, “*advocacy and training on HSS concepts and approaches to national and international stakeholders*.” Creative and ambitious activities such as this may increase the likelihood of sustainability of any program, disease-specific or otherwise.

### Financing

The Financing *building block* received only 1% of the investments in HSS. ‘Patient and/or provider incentives’ make up the vast majority of this 1% (at 93% (US$ 4 million) of the Financing *building block*). This *function*, which serves to increase patient adherence and health worker compliance thereby overcoming inequities in affordability and distribution of health services, includes *interventions* such as conditional cash transfers, pay-for-performance schemes, and remuneration for geographically isolated service providers. Specific examples include incentive cards for high-risk groups to receive Voluntary Testing and Counseling services, support for bednet voucher schemes, and incentives for community workers, healthcare providers, and laboratory technicians.

Inevitably there were examples that could only be ascribed to the second-tier. An example that fell within this *function* but did not fall specifically within the boundaries of the listed *interventions* is in a Liberian grant, “*Facilities quarterly performance award*”. Almost half of the funds dedicated to ‘patient and/or provider incentives’ were earmarked for remuneration of geographically isolated service providers (46%). Remuneration is one of the most fundamental influences on retention and redeployment of health workers to rural areas [65-67].

‘Improving resource effectiveness’ received 6% (US$ 237 000) of the funds directed towards strengthening the Financing *building block*. This *function* includes the use of evidence to plan and budget, standardization of service provider payment methods, and the support of Sector-Wide Approach (SWAp) schemes. The only activities that were funded in this *function* fell outside the boundaries of the aforementioned *interventions*. One such example is from a Sudanese grant, “*Workshop to inform the review of alternate resource generation mechanisms*”.

There is negligible funding provided to ‘maximizing social protection’ or ‘financial management transparency’. Sufficient funding dedicated to ‘maximizing social protection’ could mitigate the negative effects of multiple financing arrangements on national health financing system. This fragmentation of financing makes resource pooling virtually impossible [68]. There is a clear need to support *interventions* to improve ‘financial management transparency’. If there is such little transparency in the financial management of diligently monitored external funds, then the national health financing system is likely to be even more convoluted and leaky.

### Information

If managed improperly, GHIs can be the cause of multiple potential burdens on the national HIS of recipient countries. For example, performance-based funding can lead to the distortion of information/selective reporting and separate disease-specific information systems [69,70]. There is a growing effort of donors to harmonize and align monitoring and evaluation efforts with one another [69-73], but GHIs still demand special reporting [74]. For example, in Cambodia, Cameroon, and Uganda, the Global Fund’s project-related monitoring tools reportedly undermined the national programs [71,75]. The parallel M&E systems set up by GHIs drain time, money, and workers from the existing system though additional reporting requirements [3,74] and contribute to avoidable transaction costs [76]. As awareness of these detrimental effects has increased, efforts of GHIs to match and standardize national HIS indicators have improved [71,75-79]. There is also evidence that although GHIs are beginning to invest more in local capacity building for data management and reporting, in the development of HIS, and in technical assistance, they are still neglecting to strengthen the existing national HIS [80,81].

Although the greatest portion of Information funds was dedicated to ‘health information system strengthening, ’ *per se*, there was little variation in interventions supported within the *building block*. Only five activities were funded in three countries. Liberia received funds to expand the data collection database management and reporting system; Nigeria was funded to advocate to local government authorities and community leaders on the importance of data generation, feedback and use; and Sudan proposed to develop a data dictionary and design a survey for collecting data on the health system’s performance.

### Human resources

The Human Resources *building block* received around 23% (US$ 82 million) of the total funds dedicated to HSS in Round 8. A strong and appropriately distributed health workforce is a critical factor for expanding service coverage to improve population health [82,83]. The WHO estimates that there is a gap of close to 4 million trained healthcare workers, and Africa alone needs about 1.5 million workers trained to compensate for the deficit [84].

Around 56% (US$ 46 million) of funds for Human Resources were for continuing education/on-the-job training and 27% (US$ 23 million) to leadership and management capacity building. 10% (US$ 8 million) was dedicated to the improvement and feedback of supervision. All three of these *interventions* are ‘support for in-service health workforce’. Continuing education/on-the-job training, of course, included training for specific procedures and training of counsellors, etc., but other examples included training staff on cohort studies and procedures, training for advocacy, training in nutritional advice, training of trainers, and training of primary health care workers for quantification and forecasting for malaria.

Imbalance in investments for training for the in-service workforce is not unique to the Global Fund as supported in the primary literature. GHIs tend to focus on training in-service workers on technical areas of disease-specific concern with less attention dedicated to system-wide topics such as management and capacity building [85,86]. Few GHI resources have been allocated for pre-service support or other measures that would increase the absolute numbers of health workers [36]. Developing strong and comprehensive curricula for training pre-service workers will likely lead to strong care providers, but there then must be systems in place to retain these well-trained workers [87]. These *interventions* are interdependent and simultaneously strengthening more than one *function* will have synergistic effects.

One example of an activity that was classified as ‘support for pre-service training’, but did not fall within the boundaries of any of the *interventions* relates to a grant in Afghanistan which allocated resources to “*development of a system for conducting examinations and accreditation of the students*”. There were also some interesting activities classified as ‘support for in-service worker health workforce’: 1) development of a “*capacity needs assessment and strategic plan for the development of medical associations*”, and 2) “*contribution to HR retentions scheme*” (Liberia and Zimbabwe, respectively).

### Medicines and technology

The Medicines and Technology *building block* deals largely with the accessibility, affordability, acceptability, and availability of medicines [88]. Over the past decade access to medicines for HIV/AIDS, tuberculosis, and malaria has improved in several countries, but availability and affordability of other essential medicines remains inadequate [89]. Besides insufficient medicine supply chain and procurement systems, many country health systems are burdened by irrational use of available medicines, medical supplies, and laboratory reagents [90]. A great deal of financial support is channelled to the Medicines and Technology *building block*, but this funding should also address country-specific weaknesses relating to access of essential medicines and technologies when designing a plan of action [91].

As with some of the other *building blocks*, the magnified resolution provided by the analysis using the HSS Funding Assessment Framework shows the majority of funds dedicated to Medicines and Technology are concentrated in few activities- 53% (US$ 54 million) of the *building block*’s support was allocated to the provision/maintenance of equipment and 26% was to equip central and regional depots. Although all of the activities found in the HSS Funding Assessment Framework are important, some have potentially greater effects on the system.

Of all the *interventions* nested within Medicines and Technology, it is vital that the strengthening of supply chain management and procurement systems is not overlooked [92]. Functioning supply chain and procurement systems are critical for equitable availability, affordability, and acceptability of essential medicines [93] Examples of recipient prioritization of include the Solomon Islands’ request for funds to assess the second-level medical stores’ capacities, risks, needs, gaps, and strengthening requirements; Sudan’s development of a framework for synergistic operation of the Central Medical Stores and donor-funded procurement; and the development of standards and specifications for warehouses based on capacity needs in Liberia.

### Service delivery

The ‘infrastructure’ *function* received approximately half of Service Delivery funds, and maintenance of institutions/dispensaries was the highest funded *intervention* at 20% (US$ 23 million) of total Service Delivery spending. Maintenance of storage facilities and capital construction were not well-supported (at 5% (US$ 3 million) and 3% (US$ 2 million); respectively). Perhaps there was more money dedicated to the maintenance of storage facilities than the analysis shows; the activities could have been nested within support for institutions/dispensaries. The level of detail in the description of each activity varied, and finer differences, such as the target of maintenance, could have easily been left out of the description. Capital construction is an unlikely investment for agencies such as the Global Fund due to the lengthy, resource consuming processes it involves. Every three years the Global Fund meets with international donors for the replenishment of its funds. Therefore, it seems likely that it would prefer to invest in shorter-term activities that have more measurable results. Maintenance of institutions/dispensaries could serve as a substitute for capital construction.

‘Measures to increase coverage’ from the supply side are severely under-represented at 7% (US$ 7 million) of the *building block*. This includes *interventions* such as the standardization of care, integration of services, and support for a referral system. Of these three, the integration of services received more attention which is in line with discussions on vertical programming and HSS [6,25,28,32,73,74,94]. Despite the attention of global research community, only 5% (US$ 6 million) of funding for Service Delivery was dedicated to this *intervention*.

### HSS overall

The majority of HSS funds are dedicated to disease-specific interventions; this is in accordance with the Global Fund’s ‘diagonal’ approach to HSS – strengthening the national health system using concretely-targeted interventions [7]. There is also evidence of significant system-level support without regard to any of the three diseases, and this fact has been largely neglected in the literature. It would be interesting to compare these results to the investment profiles of other Rounds to address a number of questions. Has the percentage of system-level support increased over time? Is this related to increased understanding of systems thinking within the Global Fund Secretariat, among the applicant countries, Country Coordinating Mechanisms, and the consultants and technical agency staff who write Global Fund proposals in many countries? It would also be of interest to compare the HSS funding contained in the *approved* grants versus the *rejected* grants. Is the Global Fund more likely to fund programs that include disease-specific or system-level HSS objectives?

As discussed, within the portion of resources allocated to HSS, the activities can be further divided into the WHO-defined building blocks. It is immediately apparent that the majority of activity is within the three more ‘concrete’ *building blocks*- Human Resources, Medicines and Technology, and Service Delivery, as these three categories are easier to measure in terms of need, outcomes, and performance. On the other hand, the results achieved through investments in Governance, Financing, and Information are inherently much more difficult to evaluate; of course, within in each there are concrete *functions* and *interventions*. As discussed in the previous sections, the concrete *interventions* were more likely to be funded rather than the more complex, yet arguably more important, *interventions* operational at the interface of the building blocks. Based on the evidence of this phenomenon, it can be presumed that donors and recipients are both likely to feel more comfortable with ‘concrete’ investments, especially in terms of the performance-based funding approach of the Global Fund.

A system-wide approach results in synergistic improvements in the system with perhaps a greater balance amongst all six building blocks [9]. It is widely acknowledged that the governance and health financing systems of LMIC are relatively weak, and this is mirrored in the lack of funding for interventions in these building blocks. Greater awareness, by both funders and recipients, is required for the intervention innovation necessary for strengthening. GHIs should partner with members of the academic community to develop a book on best practices which promotes operational interventions across the health system and emphasizes the potential returns of investment in the largely neglected building blocks.

It would be interesting to perform this analysis on *Phase II* of Round 8. Are the ‘concrete’ *building blocks* still over-represented, or have the recipients shifted their focus to Governance, Financing, and Information once they are less accountable for the immediate, concrete results needed when requesting a continuation of funds and concerned more with developing sustainable health systems?

## Conclusions

This study addresses concerns in the research and development community that the Global Fund does not sufficiently contribute resources to health systems strengthening. Our results show a substantial portion (approximately 37%; more than US$ 460 million) of the Global Fund Round 8 funds were devoted to HSS, and of this, 38% (approximately US$ 140 million) was dedicated to generic system-level interventions and 62 percent (approximately US$ 223 million) dedicated to system-level interventions for the target diseases. The Service Delivery, Medicines and Technology, and Human Resources *building blocks* received the most support with 31, 28, and 23% of the HSS funds (US$ 112 million, 101 million, and 82 million); respectively. Information, Governance, and Financing *combined* received 18% of the HSS funds (12, 5, and 1% (US$ 44 million, 19 million, and 4 million) ; respectively). Within each *building block* there was significant skewing towards only one or two major *interventions*.

Furthermore, this study highlights that the Global Fund finances a diverse set of HSS activities among recipients, even within a *building block*. The lack of activity-prescription by the Global Fund allows for more personalized and creative interventions. But the dramatic skewing among the *building blocks* suggests that the Global Fund needs to explicitly define what they are willing to fund within the Governance, Financing, and Information *building blocks*. Either the request for funds in these areas is rejected by the Global Fund, or awareness by the Recipients is lacking. Either way, the Global Fund, and potentially other GHIs providing HSS funding, needs to explicitly defined interventions that address these gaps while adhering to their mandate.

Of utmost importance as we move towards the deadline for the Millennium Development Goals, is the *consensus* of international donors upon the meaning of health system strengthening in the context of aid organizations. Although the discrepancies in approach and definition provoke continued reappraisal, crucial to scholarly and policy discussion, a clear definition will help better inform funding allocations by GHIs. The community needs also to decide how to harmonize efforts *among* agencies and with countries. There should be open communication between the recipient country and all donors to enhance the potential synergies between system-level interventions. Ideally these discussions will then also lead to an agreed-upon framework with which to evaluate HSS efforts across GHI boundaries.

There is also a need for agreement, by researchers, recipients, and donors, on keystone interventions that have the greatest system-level impacts for the cost-effective use of funds. This and other retrospective studies are most useful when utilized for determining past efforts and future directions. It is necessary to understand the patterns of HSS spending within each recipient country when deciding how to proceed. Perhaps many have focused enough effort on their health sector infrastructure, e.g. transportation, technology and equipment, and modernizing facilities, to safely divert their attention to strengthening systems for which the infrastructure was developed. There is always space for creativity when developing and implementing system-level interventions; with this comes risk but even greater potential rewards.

Reaching the Millennium Development Goals requires an intensified focus on strengthening health systems. Effective health system strengthening depends on inter-agency consensus and country commitment along with concerted partnership.

## Abbreviations

EDP: Essential drugs programme; GAVI: Global alliance for vaccines and immunisation; GHIs: Global health initiatives; HIS: Health information system; HMIS: Health management information system; HSS: Health systems strengthening; IQR: Interquartile range; M&E: Monitoring and evaluation; MDGs: Millennium development goals; PEPFAR: US president’s emergency plan for AIDS relief; SWAp: Sector-wide approach; TB: Tuberculosis; WHO: World health organization.

## Competing interests

Although two authors, GS and RA, are/have been employed by the Global Fund to Fight AIDS, Tuberculosis and Malaria, neither author was involved in data analysis. They provided the data along with insight for discussion. Up to 2011, KW provided Local Fund Agent services to the Global Fund.

## Authors’ contributions

DdS, RA, and GS conceived the study. AW, DdS, and KW developed the research questions. AW and DdS developed the HSS Funding Assessment Framework. GS and RA provided access to Global Fund data and, along with AW and DdS, contributed to methodological design. AW prepared the manuscript; DdS, KW, GS, and RA edited the manuscript. All authors read and approved the final manuscript.

## Supplementary Material

Additional file 1: Table S1 HSS Funding Assessment Framework (Full). Description: Unabridged version of HSS Funding Assessment Framework. **Table S2.** Swiss TPH subset of Global Fund Round 8 grants. Description: Details of 52 grants analyzed including; country, region, disease, grant number, budgeted amount for *Phase I* (USD). **Table S3.** Distribution of Global Fund Round 8 grants by region. Description: Results of chi-squared analysis for the distribution of Global Fund Round 8 grants by region. **Table S4.** Distribution of Global Fund Round 8 grants by component. Description: Results of chi-squared analysis for the distribution of Global Fund Round 8 grants by disease component. **Table S5.** Exclusion / Inclusion criteria for health systems strengthening activities. Description: Full list of exclusion / inclusion criteria used in analysis. **Table S6.** Euro to USD exchange rates by grant start date. Description: Data for euro (€) to US Dollar ($) conversion including: grant number, grant amount (€), grant start date, daily exchange rate, grant amount ($). Click here for file
